# Phytotoxins Produced by Fungi Associated with Grapevine Trunk Diseases

**DOI:** 10.3390/toxins3121569

**Published:** 2011-12-20

**Authors:** Anna Andolfi, Laura Mugnai, Jordi Luque, Giuseppe Surico, Alessio Cimmino, Antonio Evidente

**Affiliations:** 1 Dipartimento di Scienze del Suolo, della Pianta, dell’Ambiente e delle Produzioni Animali, Università di Napoli Federico II, Via Università 100, Portici I-80055, Italy; Email: alessio.cimmino@unina.it (A.C.); evidente@unina.it (A.E.); 2 Dipartimento di Biotecnologie Agrarie, Sezione Protezione delle piante, Università degli Studi di Firenze, P.le delle Cascine 28, Firenze I-50144, Italy; Email: giuseppe.surico@unifi.it; 3 Departament de Patologia Vegetal, IRTA, Ctra. de Cabrils km 2, Cabrils E-08348, Spain; Email: jordi.luque@irta.cat

**Keywords:** black dead arm, Botryosphaeria canker, esca, Eutypa dieback, eutypiosis, grapevine trunk diseases, phytotoxins, *Phaeomoniella*, *Phaeoacremonium*, *Fomitiporia*

## Abstract

Up to 60 species of fungi in the Botryosphaeriaceae family, genera Cadophora, Cryptovalsa, Cylindrocarpon, Diatrype, Diatrypella, Eutypa, Eutypella, Fomitiporella, Fomitiporia, Inocutis, Phaeoacremonium and Phaeomoniella have been isolated from decline-affected grapevines all around the World. The main grapevine trunk diseases of mature vines are Eutypa dieback, the esca complex and cankers caused by the Botryospheriaceae, while in young vines the main diseases are Petri and black foot diseases. To understand the mechanism of these decline-associated diseases and the symptoms associated with them, the toxins produced by the pathogens involved in these diseases were isolated and characterised chemically and biologically. So far the toxins of only a small number of these decline fungi have been studied. This paper presents an overview of the toxins produced by the most serious of these vine wood pathogens: Eutypa lata, Phaeomoniella chlamydospora, Phaeoacremonium aleophilum and some taxa in the Botryosphaeriaceae family, and examines how these toxins produce decline symptoms. The chemical structure of these metabolites and in some cases their vivotoxin nature are also discussed.

## 1. Introduction

Grapevine trunk diseases cause a slow decline and yield loss in grapevines at all stages of growth. The symptoms of these diseases include the death of spurs, arms, and cordons, and the eventual death of the vines due to a progressive wood necrosis and decay of plant tissue [1,2,3,4,5,6]. Besides causing various types of wood necrosis and decay, most of these diseases also produce leaf symptoms, which include chlorosis, necrosis, deformation, and stunting.

Many fungi are involved in grapevine trunk diseases. They include species in the Botryosphaeriaceae, which cause dieback and canker, *Eutypa lata*, the agent of Eutypa dieback or eutypiosis, species of *Cylindrocarpon* and *Campylocarpon*, causing black foot disease, *Phaeomoniella chlamydospora* and *Phaeoacremonium aleophilum*, the main agents of the vascular diseases within the esca complex, including Petri disease, and species of *Fomitiporia*, *Fomitiporella*, *Inocutis* and other basidiomycetes, causing wood decay [1,3,4,5,6,7]. Recent research has established that, besides *E. lata*, some other species also in the family Diatrypaceae likewise cause trunk diseases of grapevine. These are *Eutypa leptoplaca*, *Cryptovalsa ampelina*, *Cryptosphaeria pullmanensis*, and some further species in the genera *Diatrype*, *Diatrypella* and *Eutypa* [8,9]. Most of these fungi grow slowly in the vine wood, and foliar symptoms do not appear until several years after the onset of infection, so that by the time the symptoms become visible the fungi are well-established [2,6,10].

In addition, pathogenicity has been confirmed recently for some less well-known fungi such as *Cadophora luteo-olivacea* [11,12], but many characteristic factors regarding the pathogenicity of these fungi and their mechanism of action remain to be investigated.

There is no effective cure for grapevine trunk diseases, but some remedial practices on affected vines are possible; these include the removal of dead vine parts, and re-training or re-grafting affected vines [13,14]. Moreover, the application of chemical and biological protectants on pruning wounds has been recommended to reduce the spread of these diseases [15,16,17,18,19]. Some grapevine trunk pathogens can also infect the grapevine material used in the propagation process of vines. An integrated management program including hot water treatment of vine propagation material, and the application of chemical pesticides and/or biocontrol agents at different growth stages of new vines have shown some interesting results in controlling these diseases [20,21,22].

Several pathogens involved in grapevine trunk diseases produce toxic metabolites. Many of these metabolites have been chemically characterised and tested for their toxicity on the protoplasts, calli and leaves of various *Vitis* species and on *V. vinifera* cultivars. The mode of action of some of these metabolites is well documented. This review examines the role of these toxic metabolites, the factors that ensure their virulence and their mode of action.

## 2. Eutypa Dieback

**Figure 1:**
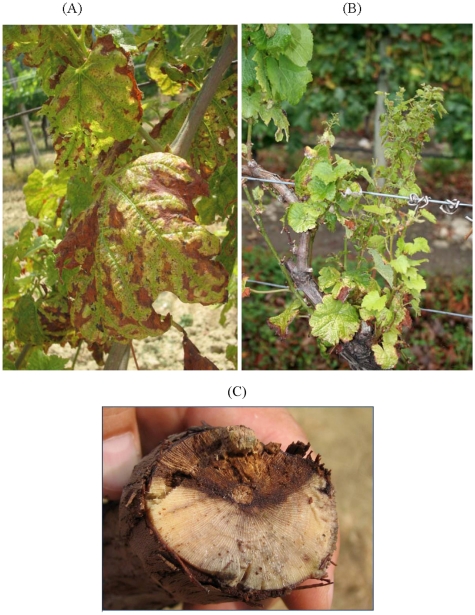
Symptoms of Eutypa dieback in (**A**) vine leaves and (**B**) new vine shoots; (**C**) Characteristic V-shaped necrosis of a vine trunk.

*Eutypa lata* causes Eutypa dieback, a serious disease that has been known for over 60 years. It causes losses in older vineyards. Its most recognised symptoms are: stunting of new shoots early in the growing season, small, cupped, chlorotic and tattered leaves, and short internodes (Figure 1A,B). Internal wood symptoms when arms and trunks are cross-sectioned include characteristic V-shaped necrosis (Figure 1C). External cankers developing from old pruning wounds also occur [1,23]. 

### 2.1. Toxins Produced by *Eutypa lata*

**Figure 2:**
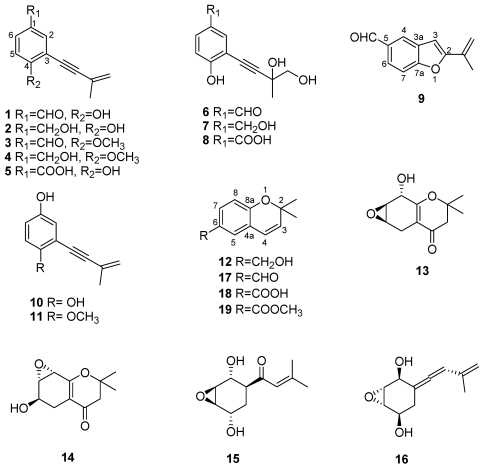
Chemical structures of *Eutypa lata* metabolites and derivatives: eutypine, eutypinol, *O*-methyleutypine, *O*-methyleutypinol, eutypin carboxylic acid analogue (**1-5**), 3-(3,4-dihydroxy-3-methyl-1-butynyl)-4-hydroxy-benzaldehyde, 2-(3,4-dihydroxy-3-methyl-1-butynyl)-4-hydroxymethyl-phenol, 3-(3,4-dihydroxy-3-methyl-1-butynyl)-4-hydroxy-benzoic acid (**6-8**), 2-*iso*-propenyl-5-formylbenzofuran, siccayne, eulatinol (**9-11**), eulatachromene and its derivatives (**12 and 17-19**), epoxidised chromanones (**13-14**), eutypoxide B and allenic epoxycyclohexane (**15-16**).

*Eutypa lata* produces a number of structurally related secondary metabolites, mainly acetylenic phenols and their heterocyclic analogues. Eutypine (**1**, Figure 2), or 4-hydroxy-3-(3-methylbut-3-ene-1-ynyl)-benzaldehyde, is the most toxic. It was isolated from the cultures of an unspecified strain of *E. lata* and characterised [24,25]. It has an unusual five-carbon acetylenic side chain. Some other, structurally related metabolites were isolated from organic extracts of the same fungal culture such as eutypinol, *O*-methyleutypin, *O*-methyleutypinol, eutypin carboxylic acid analogue (**2-5**, Figure 2), as well as the compounds produced by hydroxylation of the terminal vinyl group of eutypine, eutypinol and the carboxylic anologue (**6-8**, Figure 2) [25,26,27]. Under low acidic conditions, eutypine is converted into 2-*iso*-propenyl-5-formylbenzofuran (**9**, Figure 2). This bicyclic product was also detected in *E. lata* culture filtrates [26].

Biological assays on excised tomato plants and vine leaves have shown that of all these metabolites eutypine is the most phytotoxic [26]. A comparative study was carried out on the metabolites from three strains of *E. lata*: strain E120 from grapevine in California, strain E125 from grapevine in Italy, and isolate E178 from oak in California. The metabolites were grown on malt yeast broth (MYB) and on potato dextrose broth (PDB) [27]. Eutypine, occurred in only one of the strains but the main metabolite was eutypinol, which is a detoxification product of **1** (Figure 2) [27]. A novel metabolite, named eulatinol (**11**, Figure 2) was isolated from the Italian strain E125, together with its *O*-demethyl derivate, siccayene (**10**, Figure 2). The chromene analogue, 6-hydroxymethyl-2,2-dimethyl-2H-chromene, named eulatachromene (**12**, Figure 2) as well as **1** and **2** (Figure 2) were isolated from the California strain E120 [18]. As previously reported, eutypine was readily cyclised into benzofuran (**9**, Figure 2) in the presence of traces of acid, and eutypinol was the main metabolite under most culture conditions [27].

**Figure 3:**
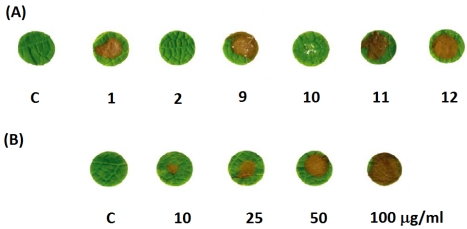
(A) Comparison, after 24 h and at 50 μg/mL, of the toxicity of the methanol control (C) and of the primary metabolites eutypine **1**, eutypinol **2**, 2-*iso*-propenyl-5-formylbenzofuran **9**, siccayene **10**, eulatinol **11**, and eulatachromene **12**, in a grape leaf bioassay; (**B**) comparison, after 24 h and at 10, 25, 50 and 100 μg/mL, of phytotoxicity of the methanol control (**C**) and eulatachromene (**12**) (redrawn with modifications from Figure 5 in [28]).

The phytotoxicity of *E. lata* metabolites was tested in a leaf disk bioassay on “*Cabernet Sauvignon*” leaves (Figure 3A) [28]. Activity at 50 μg/mL of metabolites **1, 2, and 9-12** (Figure 2) indicated that neither eutypinol nor siccayne were phytotoxic; while eutypine, benzofuran, eulatinol, and eulatacromene (**1, 9, 11 and 12**, Figure 2) had a toxic effect, producing necrotic spots on the leaves (Figure 3A). Eulatacromene and benzofuran were more toxic than the acetylenic phenols. However eulatachromene (**12**, Figure 2) was also examined for toxicity at various concentrations from 10 to 100 μg/mL. At its lowest concentration, **12** (Figure 2) caused necrosis on some disks, and at its highest concentration it caused necrosis on all disks (Figure 3B) [28].

Two highly oxygenated cyclohexene oxides were also isolated from *E. lata*: eutypoxide B [29] and the novel allenic epoxycyclohexane (**13 and 14**, Figure 2) [25]. 

To elucidate the biochemistry of *E. lata*, both its sterol composition and its total sterol content were investigated in solid and in liquid culture [30]. The total amount of sterols was 1.58 and 1.12 μg/mg dry weight mycelium in solid and liquid cultures respectively. The major sterol was identified as ergosterol. This accounted for 88% of the total sterols in a solid medium and 78% when the fungus was grown in a liquid medium. In addition to ergosterol, four minor sterols, ergosta-5,7,9(11),22-tetraen-3β-ol, ergosta-7,22-dien-3β-ol, fecosterol and episterol also occurred. They accounted for 1-4% and 1.8-13% in solid and liquid culture respectively. These results suggest that the sterol biosynthesis pathway of *E. lata* is very similar to that of other pathogenic filamentous fungi [30].

### 2.2. Structure-Toxicity Relationship Studies

Preliminary studies on the structure-toxicity relationship, carried out on the acetylenic phenols, showed that the occurrence of the aldehydic group and a free OH group in *para*-position were important for phytotoxic activity [26]. 

**Figure 4:**
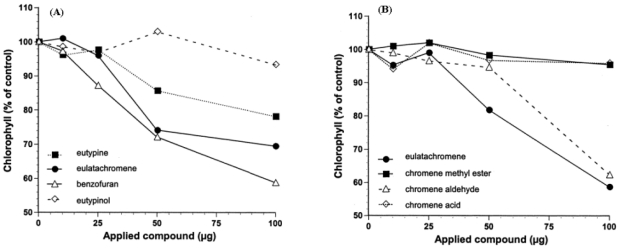
Toxicity of metabolites of *Eutypa lata* and their synthetic analogues measured in the grapeleaf disk bioassay as a per-cent reduction in chlorophyll relative to the control. (A) *E. lata* metabolites eutypine, eutypinol, 2-*iso*-propenyl-5-formylbenzofuran, siccayne and eulatachromene (**1, 2, 9, 10 and 12**, Figure 2). (B) Eulatachromene and its synthetic analogues 6-carboxymethylchromene, 6-formylchromene, and 6-carboxychromene (**12, 17-19**, Figure 2) (redrawn with modifications from Figure 5 in [31]).

Smith *et al.* [31] in their study of the structure-phytotoxicity relationships compared the activity of metabolites from various strains of *E. lata* and from their chromene analogues, such as the corresponding aldehyde, acid and methyl esters (**17-19**, Figure 2) [31]. They synthesised the chromene analogue in quantities sufficient for evaluation, and carried out a rapid quantitative bioassay, involving the topical application of individual compounds to culture disks of grape leaves with subsequent measurement of chlorophyll loss to measure tissue damage (Figure 4A,B) [31]. In this assay, eulatachromene was more phytotoxic than eutypine (Figure 4A). The bicyclic product **9** (Figure 2) was also quite toxic, while the reduction product eutypinol, as well as the quinol siccayne (Figure 4A), were not toxic [31]. When eulatachromene was compared with its three synthetic analogues **17-19** (Figure 2) only the corresponding aldehyde **17** (Figure 2) had a toxicity similar to **12** (Figure 2), while **18 and 19** (Figure 2) were not toxic (Figure 4B) [31]. Consequently the active chromenes **12** and **17**, along with benzofuran **9** (Figure 2), were more toxic than eutypine and eutypinol [31]. 

### 2.3. Mechanism of Action of *Eutypa lata* Toxins

The biological action of eutypine (**1**, Figure 2) in grapevines has been extensively studied [32]. Eutypine is synthesised by the fungus in the trunk and is thought to be transported by the sap to the herbaceous parts of the plant, where it spreads through the leaves and inflorescences to participate in the expression of disease symptoms [33]. Eutypine has weak acid properties and a marked lipophilic character. It penetrates the vine cells by passive diffusion and accumulates in the cytoplasm due to an ion-trapping mechanism related to the ionisation state of the compound [33]. In this case the experiments were carried out on the plasmalemma of three models: *V. vinifera*, *Beta vulgaris* and *Minosa pudica*. These biological models were suitable for particular physiological processes. The observations obtained from these studies showed that eutypine was unspecific since it indifferently acted on all three plant cell models used [33] (Table 1).

**Table 1:** Metabolites isolated from *Eutypa lata* and phytotoxicity assays.

Metabolite (Figure 2)	Plant matrix used for phytotoxicity assays	References
Eutypine (**1**)	Grapevine leaves, tomato leaves, grapevine, mimosa and sugar beet membranes, mitochondria	[26,27,28,31,33,34,35]
Eutypinol (**2**)	Grapevine and tomato leaves	[26,27,28,31]
*O*-Methyleutypine (**3**)	Grapevine and tomato leaves, mitochondria	[26,34,35,36]
*O*-Methyleutypinol (**4**)	Grapevine leaves, tomato leaves	[26]
Eutypin carboxylic acid anologue (**5**)	Grapevine leaves, tomato leaves	[26]
3-(3,4-Dihydroxy-3-methyl-1-butynyl)-4-hydroxy-benzaldehyde (**6**)	Grapevine leaves, tomato leaves	[26]
2-(3,4-Dihydroxy-3-methyl-1-butynyl)-4-hydroxymethyl-phenol (**7**)	Grapevine and tomato leaves	[26]
3-(3,4-Dihydroxy-3-methyl-1-butynyl)-4-hydroxy-benzoic acid (**8**)	Grapevine leaves, tomato leaves	[26]
2-*iso*-Propenyl-5-formylbenzofuran (**9**)	Grapevine and tomato leaves	[26,28,31]
Siccayne (**10**)	Grapevine leaves	[28,31]
Eulatinol (**11**)	Grapevine leaves	[28]
Eulatachromene (**12**)	Grapevine leaves	[28,31]
Epoxidised chromanones (**13, 14**)	Grapevine leaves	[26]
Eutypoxide B (**15**)	Grapevine leaves	[29]
Allenic epoxycyclohexane (**16**)	Grapevine leaves	[29]

That eutypine targets the mitochondria is suggested by the fact that it modifies the rate of respiration of the grapevine cell and its energy balance [34,35,36].

The molecular mode of action of eutypine at the mitochondrial level, and of *O*-methyleutypine (**3**, Figure 2), the non-deprotonatable derivative of **1** (Figure 2), was investigated [34]. The effects of these molecules on mitochondrial respiration and on the membrane potential were compared using isolated mitochondria from grapevine cells in suspension cultures. Eutypine caused marked stimulation of oxygen consumption and had a depolarising effect, while methyleutypine had a very slight effect on both the rate of oxygen uptake and membrane potential. High eutypine concentrations had a mixed effect, with a direct inhibition of electron transport and uncoupling. At concentrations below 200 mM, eutypine displayed a linear relationship between the oxidation rate and the membrane potential, similar to that of the traditional protonophore carbonyl cyanide *m-*chlorophenylhydrazone (CCCP). But unlike CCCP, eutypine induced a potential-dependent proton conductance, which was probably due to a potential-dependent migration of the dissociated form of the toxin across the membrane. It was thus concluded that eutypine uncouples mitochondrial oxidative phosphorylation and decreases the ADP/O ratio in grapevine cells by increasing proton leaks, which it accomplishes by means of a cyclic protonophore mechanism [35,36].

A further study of five mutant strains of *Saccharomyces cerevisiae* showed that eutypinol and eulatachromene (**2 and 12**, Figure 2) inhibited mitochondrial respiration in wild-type yeast, and that 2-*iso*-prenyl-5-formyl-benzofuran and siccayne (**9 and 10**, Figure 2) inhibited respiration in the strain lacking in mitochondrial respiration and vacuolar acidification. These effects of eutypinol and eulatachromene were confirmed using a strain with mitochondrial dysfunction and hypersensitivity to oxidative stress [32]. This study was not conducted on grapevine cells (Table 1).

### 2.4. *Eutyta lata* Toxins as Virulence Factors

*Eutypa lata* toxins are non-specific and appear to be important virulence factors in causing the symptoms of Eutypa dieback [35]. However, a lack of toxin-deficient mutants of the fungus, as well as the long time the fungus needs to incubate in the trunk before symptoms appeared, prevented a much-needed critical study of these toxins in grapevine. Nevertheless, it was confirmed that eutypine in grapevine cells is metabolised into the corresponding alcohol, eutypinol (**2**, Figure 2), through the enzymatic reduction of eutypine by a NADPH-dependent enzyme [37,38]. Eutypinol was not toxic to grapevine and had no protonophoric activity. The high affinity of the eutypine reductase enzyme (ERE) for eutypine indicated that ERE may play an important role in the detoxification of eutypine. It was also found that eutypinol did not have any uncoupling activity in the mitochondria [39] unlike what was seen with *S. cerevisiae* [32]. When the detoxification of leaf tissue by two genotypes of *V. vinifera* is compared, there seems to be a relationship between the susceptibility of vine cultivars to Eutypa dieback and the degree to which these vines deactivate eutypine [40]. Novel NADPH-dependent aldehyde reductase genes conferring resistance to eutypine have been reported from *Vigna radiata* [40,41]. This suggests that detoxification enhances the resistance of the vines to the toxin [39].

### 2.5. Analytical Detection of *Eutypa lata* Toxins *in Vitro* and *in Planta*

*Eutypa lata* synthesises a variety of metabolites, whose role in causing dieback is quite unclear. This role needs to be elucidated before any attempt can be made to apply these metabolites in order to diagnose *E. lata* dieback. Some studies have been carried out to determine the best growing conditions for *E. lata*, and to optimise its production of toxic metabolites in an artificial environment, especially on grapevine wood and wood extracts. Molyneux *et al*. [27] studied *in vitro* variations in the metabolite production of three *E. lata* isolates, using two growth media [malt yeast broth (MYB) and potato dextrose broth (PDB)], and a longer term, than previously reported. These researchers examined three representative strains of *E. lata*, two from grapevine (E120 and E125), and one strain from oak in California, to serve as a representative non-grapevine host species (E178). Metabolite composition and yield differed significantly between strains and between growth media, but yield usually peaked after 24-30 days [27]. Metabolites were identified by gas chromatography/mass spectrometry of their trimethylsilyl ether derivatives and analysed. This method proved to be generally applicable to all the metabolites subsequently isolated, with the trimethylsilyl derivatives well resolved [27]. Eutypine, eutypinol, siccayene and eulatinol (**1, 2, 10, 11**, Figure 2) were quantified by high-performance liquid chromatography (HPLC) in the three strains on MYB and PDB. Eutypine (**1**) was detected in only one of the strains (E125) on both media. The primary metabolite was the detoxification product eutypinol (**2**). This metabolite was produced in large quantities by one of the strains grown on PDB, but it was detected in all strains [18]. Strain E178 from oak did not produce these metabolites [27].

Subsequently, the HPLC procedure was optimised to establish the phenolic metabolite profiles of eleven *E. lata* strains grown on four growth media and to determine how the growth of these strains in these media differed from their growth on “*Cabernet Sauvignon*” vine wood and wood extracts [28]. The same technique was used to evaluate secondary metabolite production of 30 isolates of *E. lata* grown on media derived from the canes of three grapevine cultivars, two of which (Merlot and Semillom) were tolerant to *E. lata*, and one (Shiraz) susceptible [10]. Eutypine, eutypinol, *O*-methyl-eutypinol, 2-*iso*-propenyl-5-formylbenzofuran, eulatinol and eulatachromene (**1, 2, 4, 9, 11 and 12**, Figure 2) were detected in all culture filtrates. The most abundant metabolites were eutypinol and *O*-methyl-eutypinol, which were produced by 97 and 83% of isolates respectively. No correlation was found between secondary metabolite levels in the media containing ground canes from the three grapevine cultivars, and the tolerance/susceptibility of these cultivars to Eutypa dieback. In addition, no secondary metabolites of *E. lata* were detected from any isolates of other fungi commonly isolated from grapevine trunks in Australia such as *Cryptovalsa ampelina*, *Libertella* sp., *Pa. chlamydospora*, *Pm. aleophilum*, and various species of Botryosphaeriaceae and *Fomitiporia* that were grown on ground canes. This suggests that these metabolites are specific to *E. lata* [10]. To detect the secondary metabolites *in planta*, micropropagated grapevine plantlets were treated with purified and crude culture filtrates of nine *E. lata* isolates grown on MYB. The secondary metabolites 1, 2, 4, 9, 11 and 12 (Figure 2) were identified in some of the treated plantlets, but no single metabolite was detected consistently in all plantlets. Eutypinol was detected in micropropagated grapevine plantlets inoculated with *E. lata* mycelium; however, no metabolites were detected in the sap of plotted vines that had been mechanically inoculated with the pathogen [10]. 

As mentioned above, eutypinol is not toxic when applied to grapevine leaf disks [19,23]. However, this lack of toxicity does not preclude the use of this metabolite as a chemical marker, since the pathogenicity of *E. lata* is not due solely to the phenolic metabolites it produces, but also to the fact that it colonises grapevine wood and degrades the xylem. Consequently, whenever a non-toxic metabolite of *E. lata* such as eutypinol is detected in a vine, it is a positive indication of the occurrence of *E. lata* itself [28]. 

DNA-based markers to identify *E. lata* in infected vine wood have been developed [42,43]. Although identification techniques based on DNA analysis are sensitive enough, they are destructive and the particular vine portion sampled must actually contain *E. lata*; if healthy tissues from an infected vine are sampled, a false negative reading may result. However, the metabolites of *E. lata* are likely to be distributed throughout the vascular tissue and foliage of infected vines, especially in spring when the foliar symptoms are most evident. A diagnosis based on identifying such specific metabolites of *E. lata in planta* can therefore be carried out early in the season, even before the pathogen itself has spread throughout the vine. False negatives are in any case possible since foliar symptoms, and hence also toxin translocation, fluctuate from year to year on infected spurs or branches, as is common in leaf stripe disease [44].

## 3. Esca, A Complex of Diseases

**Figure 5:**
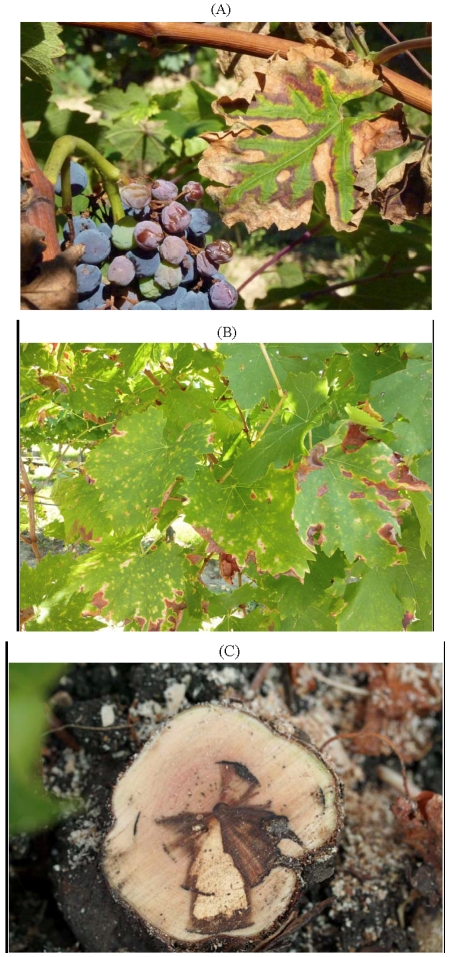
(**A**) Vine leaf showing leaf stripe disease (previously young esca) and an affected grape cluster; (**B**) leaves showing the initial interveinal chlorotic spots; (**C**) black gummy material from *Phaeomoniella chlamydospora* infected wood, brown red wood and white decay caused by *Fomitiporia mediterranea*.

The term esca complex was introduced at the end of the last century [45,46] and has more recently been redefined. The esca complex is a complex of two diseases, which may co-occur in the same vine: a wood rot (also called esca, in its redefined sense), and a vascular disease [2,47,48,49,50,51,52]. The vascular disease in turn comprises three syndromes, all of which are caused by *Pa. chlamydospora* and *Pm.* *aleophilum. Pm. aleophilum* is by far the most common [2,52] of the twenty-four *Phaeoacremonium* species that have been isolated from both symptomatic and asymptomatic grapevine wood in different parts of the world [7,48]. The three vascular syndromes are: brown wood streaking, Petri disease, and grapevine leaf-stripe disease, previously known as young esca [2,47]. Brown wood streaking is an infection of pre-planting propagation material, and produces wood discolouration and necrosis of the vessels, occurring at a stage where the vine has not yet got any leaves. Petri disease is a decline occurring in young vineyards, in which some individual vines lose their vigour and exhibit weak growth, with thin stems and a faint aspecific chlorosis or marginal necrosis of the leaves [48,49,50,51]. These symptoms are more severe in grafted than in self-rooted cuttings. The internal symptoms of visibly declining vines but also of vines that are still asymptomatic, show up as brown-black streaking of the wood in cross-section (just like brown streaking in diseased vine cuttings). Streaks often form a black ring around the pith, or appear as scattered black dots [2,52]. The symptoms of leaf stripe disease are wood necrosis (but not wood rot), brown-red discolouration, and vascular necrosis or black streaking with black gummy exudates. These symptoms are linked to the typical foliar interveinal necrosis, were the leaf blade is necrotic and is surrounded by yellow or red margins, but the area immediately surrounding the main veins remains fully green, thus giving the leaf a typical tiger-stripe pattern (Figure 5). 

In the Mediterranean area wood decay is caused almost exclusively by *Fomitiporia mediterranea*, while elsewhere it is caused by other basidiomycete species (other species of *Fomitiporia*, species of *Fomitiporella*, *Inocutis* and others) [1,3]. Symptoms are internal white decay or wood rot on artificially or naturally infected vines, but no association with any type of foliar symptom has been found. 

Lastly, since two of the diseases of the esca complex, white decay and the vascular disease, are very often found on the same vine in the field, the term esca proper also denotes a vine (especially an older vine) affected with both these diseases.

The terms chronic (or mild) esca and acute esca should therefore be abandoned. The acute form of esca indicated an apoplexy or sudden death of a vine and appears to be related to white decay, while chronic esca referred to vines showing tiger like symptoms on the leaves, which is the equivalent of what is now termed leaf-stripe disease. 

This complex of diseases causes serious losses especially in all European wine-growing countries and in the USA. While the leaf-stripe symptoms appear to be linked to the vascular disease, a sudden wilt (known as apoplexy) also occurs, which eventually leads to the death of the vine. Apoplexy occurs both in vines affected with the leaf-stripe symptoms and in vines with white rot which is itself the combined effect of on the one hand extensive wood deterioration caused by the rot fungi, and on the other hand certain toxins produced by *Pa. chlamydospora* and/or *Pm. aleophilum* [53]. The shoots, leaves, and berries also become affected (Figure 5).

To simplify the reading of this review, only the term esca will be used from now on to refer to the esca complex in general.

### 3.1. Toxins Produced by *Phaeomoniella chlamydospora* and *Phaeoacremonium aleophilum*, Main Causal Agents of Leaf Stripe Disease

#### 3.1.1. Lipophilic Low-Molecular Weight Metabolites

**Figure 6:**
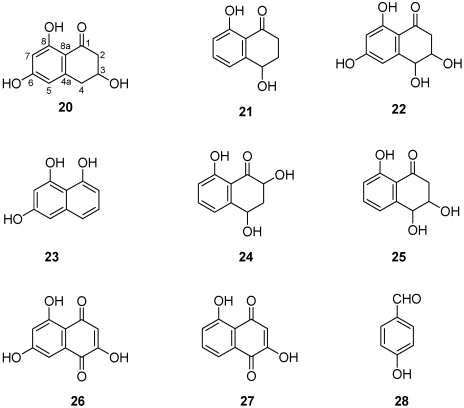
Chemical structures of *Phaeoacremonium aleophilum* metabolites: scytalone, isosclerone, *cis*-4-hydroxyscytalone, 1,3,8-trihydroxynaphtalene, 2,4,8-trihydroxytetralone, 3,4,8-trihydroxytetralone, flavioline, 2-hydroyjuglone and 4-hydroxybenzaldehyde **20-28**.

**Figure 7:**
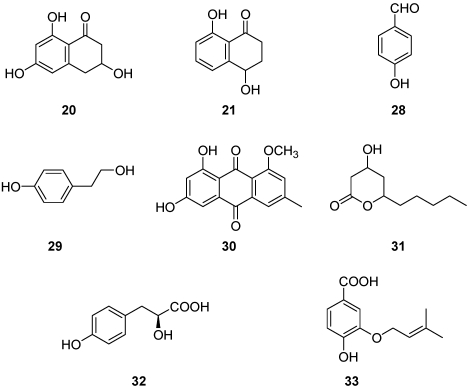
Chemical structures of *Phaeomoniella chlamydospora* metabolites: scytalone, isosclerone (**20-21**), 4-hydroxybenzaldeide, tyrosol, 1-*O*-methylemodine, 3-hydroxy-5-decanolide, (*S*)-4-hydroxyphenyllactic acid, 3-(3-methyl-2-butenyloxy)-4-hydroxybenzoic acid (**28-33**).

Various metabolites, representing different classes of natural compounds, have been isolated and identified from culture filtrates of *Pm. aleophilum* and *Pa. chlamydospora.* They include naphthalenone pentaketides. From liquid culture of *Pm. aleophilum* were isolated scytalone, isosclerone [54,55], *cis*-4-hydroxy-scytalone, 1,3,8-trihydroxynaphtalene (1,3,8-THN), 2,4,8-trihydroxytetralone (2,4,8-THT), 3,4,8-trihydroxytetralone (3,4,8-THT), flavioline, 2-hydroxyjuglone (2-HJ, traces), and 4-hydroxybenzaldehyde (**20-28**, Figure 6) [55]. Liquid cultures of *Pa. chlamydospora*, have yielded, besides scytalone, isosclerone and 4-hydroxybenzaldehyde already mentioned (compounds **20, 21, 28**, Figure 7) [55], also tyrosol, 1-*O*-methylemodine, 3-hydroxy-5-decanolide, (*S*)-4-hydroxyphenyllactic acid and 4-hydroxy-3-(3-methyl-2-butenyloxy)-benzoic acid (**29-33**, Figure 7) [55].

The biological activity of metabolites produced by *Pm. aleophilum* and *Pa. chlamydospora* were evaluated against grapevine leaves, calli and living protoplasts (Table 2).

**Table 2:** Metabolites isolated from *Phaeoacremonium aleophilum* and *Phaeomoniella chlamydospora* and phytotoxicity assays.

Metabolite (Figure 6 and Figure 7)	Plant matrix used for phytotoxicity assays	References
Scytalone (**20**)	Grapevine leaves Grapevine callus and protoplasts *Arabidopsis thaliana*	[54,55,56,57]
Isosclerone (**21**)	Grapevine leaves Grapevine callus and protoplasts *Arabidopsis thaliana*	[54,55,56,57]
*cis*-4-Hydroxyscytalone (**22**)	Grapevine callus and protoplasts	[57]
1,3,8-Trihydroxynaphthalene (1,3,8-THN) (**23**)	Grapevine callus	[57]
2,4,8-Trihydroxy-tetralone (2,4,8-THT) (**24**)	Grapevine callus *Arabidopsis thaliana*	[57]
3,4,8-Trihydroxytetralone (3,4,8-THT) (**25**)	Grapevine callus *Arabidopsis thaliana*	[57]
Flavioline (**26**)	Grapevine callus *Arabidopsis thaliana*	[55]
2-Hydroyjuglone (**27**)	Grapevine callus	[57]
4-Hydroxybenzaldehyde (**28**)	Grapevine callus and protoplasts	[55,57]
(*S*)-4-Hydroxy-phenyllatic acid (**32**)	Grapevine callus and protoplasts	[55]
3-(3-Methyl-2-butenyloxy)-4-hydroxybenzoic acid (**33**)	Grapevine callus and protoplasts	[55]

**Figure 8:**
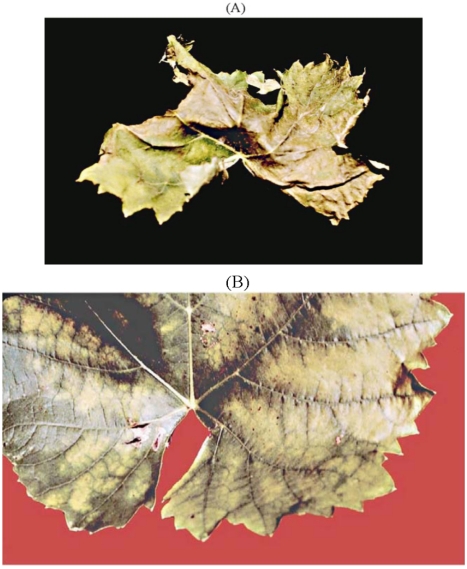
Absorption of (**A**) 3 mL of 0.05 mg mL^−1^ scytalone and (**B**) 0.1 mg mL^−1^ isoslerone by detached leaves of grapevine cv. Italia exposed to these solutions for a few hours .(Reproduced with permission from the authors of [54]).

Other metabolite activities, usually related to the natural ageing of the leaves (loss of cell membrane semipermeability-causing ion leakage-and membrane-lipid peroxidase) were also examined. Scytalone (**20**, Figure 6 and Figure 7) assayed at 0.05 mg/mL on grapevine cv. Italia leaves, produced spots that were light-green to chlorotic, round to irregular, and that became coalescent or remained spread out over the leaf blade (Figure 8A). Isosclerone (**21**, Figure 6 and Figure 7), assayed at 0.1 mg/mL, caused large yellowish spots that slowly became coalescent and necrotic, and that were followed by distortion and withering of the leaf lamina (Figure 8B) [54]. In a number of *in vitro* tests, leaf cultures from grapevine cv. Thompson absorbing 0.1 and 0.05 mg/mL solutions containing scytalone or isosclerone only showed some marginal browning, slightly stronger at the higher concentration [56]. Scytalone was tested for factors relating to leaf ageing, but no real effect was seen (scytalone caused higher peroxidation, but only in the first 30 min). On the other hand both scytalone and isosclerone increased ion leakage, and when leaf disks were soaked in a scytalone solution at a concentration of 0.05 μg/mL, membrane semipermeability was impaired.

Scytalone and isosclerone (**20-21**, Figure 6 and Figure 7) increased growth *in vitro* of calli of *V. vinifera* cv. Gamay at the lowest concentrations, 0.1 and 0.25 mM respectively. Similar increases in callus growth were achieved with 3,4,8-THT (**25**, Figure 6) at 0.1 mM. On the other hand, the naphthoquinones such as juglone, 2-hydroxyjuglone (2-HJ) and 3-hydroxyjuglone (3-HJ) inhibited callus growth at 0.1 nM, and flaviolin (**26**, Figure 6) inhibited callus growth at 0.25 mM [57]. These results differed slightly from the trials on calli from cv. Thompson and cv. Cabernet. When callus cultures from these cultivars were grown on a medium containing scytalone and isosclerone solutions at concentration increasing from 0.02 to 0.05 and 0.1 mg/mL, the callus cultures became increasingly brown going from light brown to black; however, callus discolouration did not stop or reduce callus growth on either cv. Thompson seedless or cv. Cabernet [56].

The biological activity of 4-hydroxybenzaldehyde, (*S*)-4-hydroxyphenyllactic acid and 4-hydroxy-3-(3-methyl-2-butenyloxy)-benzoic acid (**28, 32-33**, Figure 7) was determined on living protoplasts, from a hydroponic culture of *Vitis vinifera* cvs. Cabernet Sauvignon and Ugni blanc, by calculating the percentage of surviving protoplasts after growing them for 24 h together with the compounds at concentrations of from 10^−5^ to 10^−6^ M. 4-Hydroxybenzaldehyde (**28**, Figure 6 and Figure 7) caused 20% mortality of protoplasts at 100 nM and 100% mortality at 1 mM. Metabolites **32 and 33** (Figure 7) were active on the protoplasts even at a concentration of 1 mM [57] (Table 2). 

#### 3.1.2. Hydrophilic High-Molecular Weight Metabolites

#### 3.1.2.1. Polysaccharides

When *Pa. chlamydospora* and *Pm. aleophilum* were grown in liquid culture, they produced exopolysaccharides (EPSs) in some tests [58]. In recent years, the structure of a large number of EPSs has been determined due to advances in ways to purify and chemically characterise these compounds [59].

That EPSs are involved in bacterial and fungal diseases has been reported by Hogdson *et al*. (1949); Harborne (1983) and Denny (1995) [60,61], but their activity as phytotoxins still remains to be clarified [62,63]. A number of phytopathogenic fungi produced EPSs toxic to plants. These fungi are *Cephalosporium* [64], *Ceratocystis fagacearum* [65], *Ophiostoma ulmi* [66], *Fusarium solani* [67], and several species of *Phytophthora*, including *P. cinnamomi*, *P. megasperma* var. *sojae* and *P. palmivora*, pathogens of forest, ornamental and agrarian plants such as oak, juniper and soybean [67]. Moreover, EPSs from the culture filtrates of *P. cinnamomi*, *P. cryptogea* and *P. nicotianae* cause severe wilting on several hosts [68]. It is thought that these macromolecules interfere with water movement in the plant tissues by plugging the vessels, and that this causes the wilt symptoms [69]. The wilting seems to be related to the size of the molecules and their viscosity rather than to their structure [70]. However some other experimental findings on host specificity suggest that the EPSs differently act. In fact, EPSs produced by some bacteria induced leaf spot diseases only on the host plants [71]. Furthermore, EPSs recently isolated from *Cryphonectria parasitica* [72], *Phomopsis foeniculi* [73] and *Neofusicoccum parvum* [74], were toxic to both hosts and non-host plants, and this suggests that they have non-specific phytoxicity.

Preliminary investigations, essentially using HPLC, molecular analysis and infrared spectroscopy, have revealed that *Pa. chlamydospora* and *Pm. aleophilum* produce pullulan [58]. Pullulan is a polysaccharide polymer consisting of maltotriose units, also known as α-(1°4)-; α-(1°6)-glucan. Three glucose units in maltotriose are connected by an α-(1°4)- glycosidic bond, whereas consecutive maltotriose units are connected to each other by an α-(1°6) glycosidic bond. Commercially pullulan is usually produced by the fungus *Aureobasidium pullulans* [75,76].

To establish the role of EPSs in esca, the EPSs were characterised biologically and their occurrence in infected vines was determined. It was found that when EPSs produced by *Pm. aleophilum* and *Pa. chlamydospora* were absorbed at very low doses by detached grapevine leaves, or when they were injected into the woody tissue of the shoots and branches of standing grapevines, they caused foliar symptoms similar to those shown by vines with esca. The same symptoms were produced when the vine leaves were treated with pullulan extracted from the discoloured wood of a grapevine infected with *Pa. chlamydospora*, and with commercial pullulan [58].

When culture filtrates of *Pa. chlamydospora*, or weak solutions of purified preparations of scytalone, isosclerone or pullulan, were absorbed by detached leaves and berries they caused symptoms similar to those of vines affected with esca in the field [77].

#### 3.1.2.2. Polypeptide Toxic Metabolites

Luini *et al*. [78] also reported on the toxic activity of polypeptides secreted by *Pa. chlamydospora* and *Pm. aleophilum* in their culture medium. The structures of these polypeptides have not yet been determined. 

Even though the electrophoretic patterns of the polypeptides differed from those of the EPSs, their biological activity was very similar. They produced anthocyanins on grapevine leaves and, when applied to grapevine cells in culture, they modified proton fluxes, depolarised the cell membrane, inhibited the transport of sucrose and glutamine and, lastly, caused the death of the cells. Moreover, polypeptides can now be traced by immunological methods, as reported by Fleurat-Lessard *et al*. [79], and this has confirmed that these compounds have a significant role in the disease, and one that deserves further investigation.

### 3.2. Toxins Produced by *Fomitiporia mediterranea*, a Causal Agent of Wood Rot in Grapevine.

**Figure 9:**
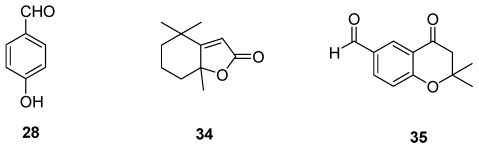
Chemical structures of *Fomitiporia mediterranea* metabolites: 4-hydroxybenzaldeide (**28**), dihydroactinolide (**34**) and 6-formyl-2,2-methyl-4-chromanone (**35**).

The toxic metabolites produced by *F. mediterranea*, the basidiomycetous fungus which, in Europe, is most frequently associated with grapevine white rot, were also investigated. Tabacchi *et al*. [55] reported that the culture filtrate of *F. punctata* (current nomenclature: *F. mediterranea*) produced 4-hydroxy-benzaldehyde (**28**, Figure 9), dihydroactinolide and a novel chromanone, called 6-formyl-2,2-dimethyl-4-chromanone, and biogenetically related to eutypine (**34-35**, Figure 9).

White [80] reported that ten basidiomycetes isolated from grapevine in South Africa (8 novel species in the genera *Fomitiporella*, *Fomitiporia*, *Inonotus*, *Inocutis*, and *Phellinus*, and two species in an undetermined genus) produced *in vitro* 4-hydroxybenzaldehyde at fairly low concentrations ranging from 0.005 to 0.06 mg/L. The level of 4-hydroxybenzaldehyde was not related to a specific taxon and varied within each taxon [80]. 

### 3.3. Mechanism of Action of Toxins Involved in the Esca Complex (Vascular Disease and Wood Rot)

The mode of action of the toxins, essentially naphthoquinones, produced by the fungi involved in esca may be related to their oxidant property. The quinones are extensively used in studies of oxidative stress because of the important role they play in plant defence [81]. Scytalone levels in particular may be related to the intensity of the brown-black colour of infected vine wood and to the occurrence of dark material in the xylem vessels.

**Figure 10:**
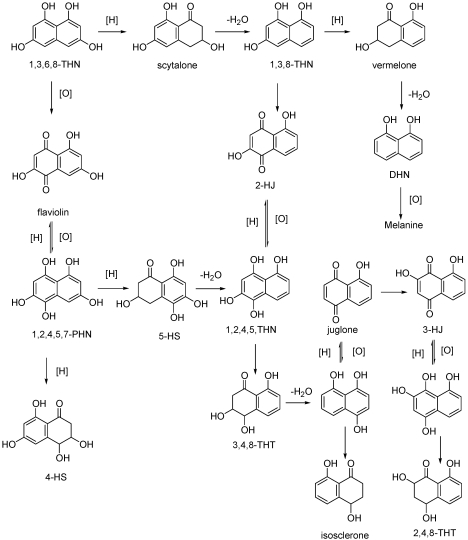
The pentaketide pathway of melanin synthesis with flavioline and 2-hydroxyjuglone (2-HJ). 1,3,6,8-tetrahydroxynaphthalene (1,3,6,8-THN); 1,3,8-tri- hydroxynaphthalene (1,3,8-THN); 1,2,4,5,7-pentahydroxynaphthalene (1,2,4,5,7-PHN); 5-hydroxyscytalone (5-HS); cis-4-hydroxyscytalone (4-HS); 1,2,4,5-tetrahydroxy- naphthalene (1,2,4,5-THN); 3,4,8-trihydroxytetralone (3,4,8-THT); 2,4,8-trihydroxy- tetralone (2,4,8-THT). (Reproduced with permission from the authors of [56])

Scytalone and the naphthoquinones produced by *Pa. chlamydospora* and *Pm. aleophilum* are intermediate metabolites resulting from the biosyntesis of dihydroxynaphthale (DHN) melanins. Some studies examined the pentaketide pathway of melanin synthesis in some black fungi pathogenic to humans and plants [82,83]. As shown in Figure 10, scytalone may be produced by the oxidation of 1,3,8-THN.

Melanins are dark pigments of a phenolic nature with a high molecular weight, which occur in animals, plants and micro-organisms. The structure of these pigments is unknown, and they probably are not necessary for the life of the producing organism, but they increase the capacity of that organism to survive under certain conditions. For example the occurrence of melanins in the cell wall of some fungi [84,85,86] increases the resistance of these fungi to physical agents such as UV radiation [86,87].

The role of melanins in virulence in may be due to various factors. Bürki *et al*. [88] reported that the naphthoquinones are cytotoxic, and they are thought to act by forming a covalent adduct through a Michael 1,4 addition between the quinone and a protein thiol or amino group [89]. Of these compounds, the most widely studied is juglone, which is highly toxic to plants and animals [61]. In fungal cells, juglone generates superoxide radicals, diminishing the cellular pool of reduced pyridine nucleotides which are particularly necessary for cells exposed to oxidative stress. It is well known that one of the main ways of plants to respond to phytopathogenic invasion is by generating O_2_^−^ and H_2_O_2_ [90,91]. Many plants also produce auto-oxidisable quinone defence compounds, which oxidise NAD(P)H in plant and pathogenic cells, leading to the formation of superoxide radicals and hydrogen peroxide [92]. In response to these attempts of the plant to defend itself, the pathogenic fungi synthesise the naphthoquinones, which are toxic and suppress the defence reaction [93].

The naphthoquinones lower the resistance of plants to pathogenic fungi and hence enhance the virulence of those fungi. The production of naphthoquinone pigments appears to be an important component of the disease process as these pigments act as non-specific virulence factors inhibiting the plant defence reaction, resulting in plant hypersensitivity [94].

It has been reported that actively melanising cell-like appressoria of *P. oryzae* produce higher levels of melanin intermediates or their derivatives (phenols and quinones) in the presence of agents blocking melanin biosynthesis such as Tricyclazole^®^ or Carpropamide^®^ [95]. As phenols and quinones are highly toxic to living cells, an abnormal accumulation of metabolites such as 2-HJ probably prevents the appressoria from functioning [96].

Not much is known about how these metabolites act in the vine cells or tissues. The physiological changes that occur in the chlorotic portion of leaves with tiger-stripes, in the surrounding green portion of these leaves, and in the asymptomatic leaves of vine arms that also bear tiger-striped leaves, indirectly suggest that these changes are caused by toxic metabolites. Physiological changes include alterations in the rate of gas exchange, chlorophyll concentration, net photosynthesis, stomatic conductance, intercellular concentration of CO_2_, and transpiration rate [97,98]. Tiger-striped leaves also show higher levels of fructose and glucose and lower levels of sucrose. Starch goes down while abscissic acid goes up, as do the free amino-acids, especially proline [98,99]. The mechanism whereby these disorders are produced has not yet been elucidated, but the changes may be an indirect effect of the toxins produced by the fungi colonising the vine wood, and perhaps even by some non-pathogenic fungi which also abundantly colonise the vine wood. In that case these changes would be a response of the vine to the disease. For example, the increase of abcissic acid in the leaves, as also the increase in soluble sugars and amino-acids, appears to be a stress-response to an impairment of the conducting vessels [100]. 

Scytalone and isosclerone also cause peroxidation of the membrane lipids, and this is known to be related to leaf senescence [56]. Toxic polypeptides could also be involved in this early senescence process, since they cause an increase in the anthocyanin levels of vine leaves [78].

### 3.4. Chemical and Immunological Detection of Esca-Related Metabolites

As a rule, esca is first detected by visual inspection of the foliar symptoms. Visual inspection is completed by isolating on a growth medium the fungal colonies growing out of a piece of infected wood tissue and by identifying the growing fungus using microscopic inspection of the mycelium and the conidia, and/or by molecular methods. 

A new means of detection is to employ HPLC or immunological techniques to identify the secondary metabolites secreted by the pathogen that is to be detected. Studies based on these techniques have established that secondary metabolites have a role as vivotoxins [101]

#### 3.4.1. HPLC Technique

The role of fungal metabolites in causing symptoms on esca-affected vine leaves and berries was studied in a vineyard of *V. vinifera* cv. Italia located in southern Italy [102]. During early spring, two to four branches per vine were pruned and samples of xylem sap were collected. Vine bleeding, assessed as mL day^−1^ vine^−1^, peaked at bud burst and stopped within 28 days. During this period, the leaf water potential of diseased vines progressively increased (*i.e.*, values became less negative), indicating a dysfunction in the supply of water and nutrients to the new growth. Fungi were isolated both from the xylem sap and from the woody tissue of the branches and trunks of diseased vines. Conidia isolated from the sap showed a high germination rate (>90%). Bioactive concentrations of the two pentaketides were detected in the xylem sap, leaves and berries at various stages of seasonal growth [58,103]. Exopolysaccharides, including pullulan, were found in the xylem sap. 

#### 3.4.2. Immunological Techniques: Flow Cytometry

**Figure 11:**
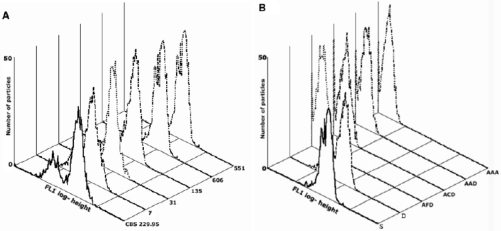
(**A**) Cytofluorimetric profiles of *Phaeomoniella chlamydospora* extracts from strains CBS 229.95, 7, 31, 135, 551 and 606. (**B**) Cytofluorimetric profile of leaf extracts of asymptomatic vine leaves from asymptomatic vine no. 61 (AAA) and from symptomatic vine no. 58. The position on the x-axis clearly indicates no recognition by the antibody-labelled beads of the extracts from leaves from asymptomatic vines, and from asymptomatic leaves from asymptomatic shoots (ADD), not even when the leaves were close (ACD) or distal (AFD) to a symptomatic leaf in a symptomatic shoot (ADD). In contrast when the extracts of leaves with incipient chlorotic symptoms or leaves with fully developed symptoms from a diseased vine were tested (samples D and S, respectively), the position of the profiles on the x-axis indicated there was strong recognition by the antibodies. Axis of abscissa is FL-1og height; axis of ordinate is number of particles. (Reproduced with permission from the authors of [103]).

**Figure 12:**
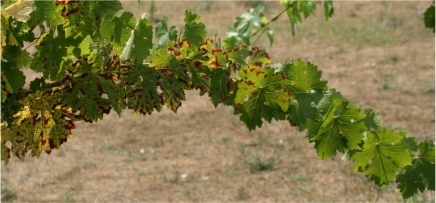
Esca-diseased grapevine leaves with tiger-stripe discoloration: a shoot showing the acropetal symptom gradient. (Reproduced with permission from the authors of [103])

A study has recently been carried out to develop a rapid and specific method for the production of polyclonal antibodies against the EPSs produced by *Pa. chlamydospora* and to detect these EPSs in the leaves of esca-affected grapevines [103]. The *Pa. chlamydospora* EPSs were unequivocally identified in diseased vines using flow cytometry. Antibodies raised against *Pa. chlamydospora* EPSs were used as an antigen to immunise rats. Their specificity was also tested against other *Pa. chlamydospora* strains (Figure 11A). The antibodies did not recognise control EPSs from *P. foeniculi* or from *P. exigua* var. *heteromorpha*, nor EPSs from *Pm. aleophilum*, *Neofusicoccum luteum* or *Neofusicoccum parvum*, indicating that these antibodies were specific to the EPSs of *Pa. chlamydsopora* [103]. Flow cytometry used *in planta* positively detected *Pa. chlamydospora* EPSs from esca-affected leaves showing interveinal and marginal discolouration, and necrosis (Figure 12) [103].

#### 3.4.3. Immunological Techniques: Serological Test

*Phaeomoniella chlamydospora* in a culture medium secretes a variety of polypeptides [104,105], the biochemical nature of which made it possible to devise a serological test to detect them [79]. Polyclonal antibodies raised in rabbit against the polypeptide fraction produced by a *Pa. chlamydospora* culture medium recognised small amounts of the secreted fungal proteins (commonly 1 ng). These antibodies had a valuable specificity because they cross-reacted with the polypeptides secreted by various strains of *Pa. chlamydospora* but not with polypeptides secreted by any of the many other fungal pathogens commonly found in fungus-infected vine wood. Importantly, as shown by the enzyme-linked immunosorbent assay (ELISA) and by immunolocalisation on ultrathin sections, they did not cross-react with the polypeptides secreted by fungi causing other types of wood decay such as *E. lata*, or fungi causing cankers such as the Botryosphaeriaceae *D. seriata* and *N. parvum*. *Pa. chlamydospora* was detected serologically in canes of mechanically infected cuttings [79]. The specificity of the antibodies was tested against fungal proteins from four strains of *Pa. chlamydospora*, since it is essential that the antibodies raised towards one strain should also be selective towards other strains of the same fungus. Western blot analysis confirmed that the antibodies directed towards the secreted proteins of strain PC-PC37 also recognised the other strains, although the response intensity differed between strains in some areas of the transferred bands (e.g., see the 98 kDa bands). The global response measured by ELISA showed that the intensity of the reaction presented slight variations depending on the origin of the secreted antigen [79]. This made it possible to detect *Pa. chlamydospora* in grapevine with a dot blot method that was simple, rapid, reliable and non-destructive of the vines tested [79].

### 3.5. NMR Metabolomics of Esca

To study the metabolic changes in grapevine plants affected with esca, leaves were analysed from both symptomatic and non-symptomatic cordons of *V. vinifera* cv. Alvarinho, collected in the Vinho Verde region, Portugal. The metabolite composition of leaves from infected cordons with visible symptoms [diseased leaves (DL)] and from asymptomatic cordons [healthy leaves (HL)] was evaluated by 1D and 2D ^1^H-nuclear magnetic resonance (NMR) spectroscopy [106]. 

Principal component analysis (PCA) of the NMR spectra showed a clear separation between DL and HL leaves, indicating that the compounds produced by these two types of leaves differed. NMR/PCA analysis identified the compounds belonging to each of the groups.

Altogether, levels of phenolic compounds were significantly higher in DL leaves than in HL leaves, and carbohydrate levels were significantly less, suggesting that the diseased leaves were rerouting carbon and energy from primary to secondary metabolism. The diseased leaves also had higher levels of methanol, alanine, and *γ*-aminobutyric acid, which may have been caused by the activation of other defence mechanisms [106]

## 4. Botryosphaeriaceae Species Involved in Grapevine Trunk Diseases

**Figure 13:**
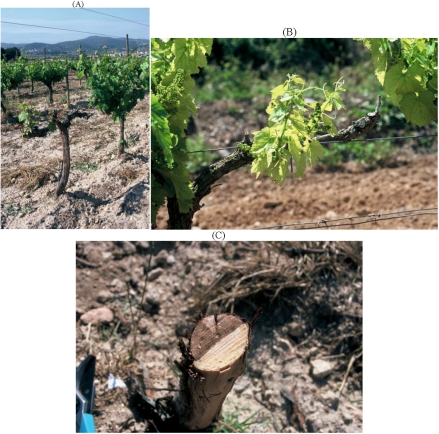
Symptoms of grapevine affected with various species of Botryosphaeriaceae. (**A**) Stunting early in the season; **B**) Foliar chlorosis; (**C**) wedge-shaped necrosis in a trunk cross-section.

In recent years an increasing number of Botryosphaeriaceae species have been associated with grapevine decline worldwide [6,107,108]. Several diseases caused by these pathogens in grapevines are known, or have been known in the past, under a variety of names such as black dead arm (BDA), *Botryosphaeria* canker, excoriose, *Diplodia* cane dieback and bunch rot [6,109,110,111,112]. External symptoms of these diseases include death of the cordons, canes, shoots and buds, canker formation, stunting, delayed bud burst, bud necrosis, bleached canes, reduced bunch set and bunch rots, all of which have been extensively documented [6,109,110,111,112,113,114,115,116]. Internal symptoms on the trunks or canes of declining vines such as brown wood streaking and wedge-shaped discolourations are commonly associated with Botryosphaeriaceae species [6,111,112,113,116]. Some of the symptoms, both external and internal, are shown in Figure 13.

### 4.1. Phytotoxic Metabolites from Botryosphaeriaceae Species

**Figure 14:**
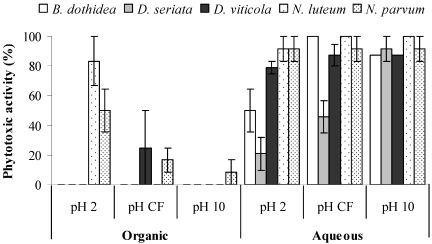
Toxic activity of organic extracts and related acqueous phases obtained from culture filtrates of *Botryosphaeria dothidea*, *Diplodia seriata*, *Dothiorella viticola*, *Neofusicoccum luteum* and *N. parvum* assayed on tomato plants. (Reproduced with permission from the authors of [74])

Five species isolated from declining grapevines in Spain, *Botryosphaeria dothidea*, *Diplodia seriata*, *Dothiorella viticola*, *Neofusicoccum luteum* and *N. parvum* were examined for toxin production in liquid cultures of Czapek-Dox broth for different lengths of time [74]. All fungi produced high-molecular weight hydrophilic compounds with toxic properties (Figure 14).

**Figure 15:**
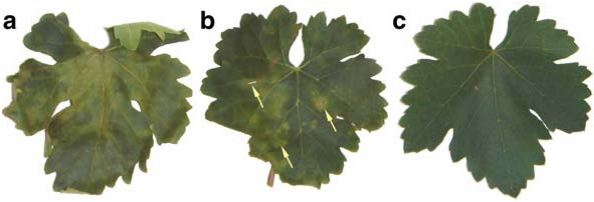
Symptoms caused on grapevine leaves of the cv. Tempranillo by 14-day-old culture filtrates of *Neofusicoccum parvum*: (**A**) severe withering; (**B**) partial withering with necrotic spots (arrows); (**C**) symptomless leaf (control immersed in distilled water). (Reproduced with permission from the authors of [74])

Toxin production of *D. seriata* and *N. parvum* peaked after 14 days in culture, while that of other species peaked after 21 days. The effect of 14 day-old *N. parvum* culture filtrate on grapevine leaves cv. Tempranillo is shown in Figure 15. 

The high-molecular weight hydrophilic toxic compounds produced by *N. parvum*, which were later identified as EPSs, were further tested. GC-MS analysis of the acetylated *O*-methyl glycosides of these EPSs revealed that these compounds consisted mainly of glucose, mannose and galactose, and that they differed from the botryosphaerans [70]. The botryosphaerans are branched β-(1→3; 1→6)-D-glucans produced by *B. rhodina* [117] and by an unidentified ligninolytic *Botryosphaeria* sp. [70]. The role of EPSs in bacterial and fungal diseases has been revised in depth [118]. In addition, *N. luteum* and *N. parvum* produce low molecular weight lipophilic phytotoxins (Figure 14), that do not invariably occur in all the remaining species [74].

The sections following deal with the toxins produced by the most familiar Botryosphaeriaceae species associated with grapevine, *N. parvum* and *D. seriata*. However, further investigation, such as that into the toxins produced by other botryosphaeriaceous fungi and that into the toxic compounds of infected grapevines, is needed to better understand the role that these and other toxic compounds play in symptom expression.

### 4.2. Toxins Produced by *Neofusicoccum parvum*

**Figure 16:**
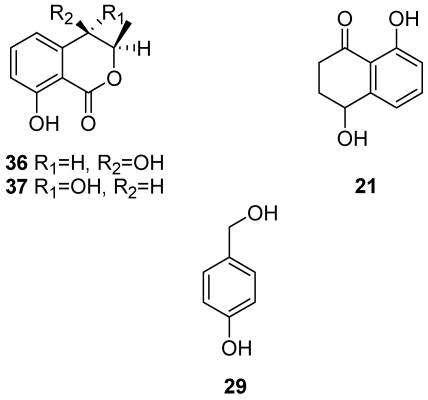
Structures of (3*R*,4*R*)-(-)-4-hydroxymellein, (3*R*,4*S*)-(-)-4-hydroxymellein, isosclerone and tyrosol (**36, 37, 21, 29**).

In a recent study has isolated the metabolites produced by *N. parvum* (strain CBS 121486) in optimised culture condition and characterised them chemically and biologically [119]. Four toxic metabolites were isolated from the organic extract and identified by spectroscopic and physical examination as (3*R*,4*R*)-(-)-4-hydroxy- and (3*R*,4*S*)-(-)-4-hydroxy-mellein, isosclerone, and tyrosol (**36, 37, 21, 29**, Figure 16), which were reported as being produced by *N. parvum* [119] for the first time.

When assayed on tomato cuttings all four metabolites (**21, 29, 36, 37**, Figure 16) were toxic, producing symptoms ranging from slight to severe leaf wilting (Table 3). (3*R*,4*R*)-(-)-4-hydroxymellein and isosclerone were the most toxic. All these toxins have already been reported as being produced by many phytopathogenic fungi [54,120,121,122,123,124,125,126,127]. 

Moreover, isosclerone (**21**, Figure 16) was reported for the first time as produced by a Botryosphaeriaceae species [119]. (3*R*,4*R*)-(-)-4-hydroxymellein (**36**, Figure 16) is produced, together with other melleins and tyrosol (**29**, Figure 16), by a strain of *B. obtusa* (syn. *D. seriata*) that causes frogeye leaf spot and black rot of apple [125,128]. The same 4-hydroxymellein as well as its steroisomer (3*R*,4*S*)-(-)-4-hydroxymellein and mellein are also produced by *Diplodia pinea* (Desm.) Kickx, another Botryosphaeriaceae species, which causes decline of *Pinus radiata* D. Don in Sardinia, Italy [127].

**Table 3:** Metabolites isolated from *Neofusicoccum parvum* and their toxicity assayed on tomato plants. (Reproduced with permission from the authors of [118])

Metabolite	Concentration (M)	Toxicity rating a			Mean toxicity rating ± S.E.
		Rep 1	Rep 2	Rep 3	
(3*R*,4*R*)-(-)-4-Hydroxymellein (**36**)	0.26 × 10^−3^	2	2	3	2.3 ± 0.33
	0.13 × 10^−3^	1	1	3	1.7 ± 0.67
	0.26 × 10^−4^	1	1	2	1.3 ± 0.33
(3*R*,4*S*)-(-)-4-Hydroxymellein (**37**)	0.26 × 10^−3^	0	1	2	1.0 ± 0.58
	0.13 × 10^−3^	0	1	1	0.7 ± 0.33
	0.26 × 10^−4^	0	1	0	0.3 ± 0.33
Isosclerone (**21**)	0.31 × 10^−3^	2	3	1	2.0 ± 0.57
Tyrosol (**29**)	1.60 × 10^−3^	2	2	1	1.7 ± 0.33
Control/Czapek-Dox		0	0	0	0.0 ± 0.00
Control/H_2_O		0	1	0	0.3 ± 0.33

^a^ Lesion symptoms were evaluated using a 0-3 scale: (0) no symptoms; (1) slight wilting in one leaf; (2) moderate wilting on some leaves; (3) severe wilting on leaves (with necrotic spots on leaves occurring occasionally). Three plants (Rep 1 to Rep 3) were used for each sample tested.

Botryosphaeriaceae species were reported by Larignon *et al*. [95] to cause foliar symptoms (*i.e.*, tiger-striped chlorosis and necrosis) but these cannot be clearly distinguished from the symptoms of grapevine leaf stripe (previously young esca). Those symptoms were described as belonging to a disease called black dead arm (BDA) which was attributed to several species of Botryosphaeriaceae. The difficulty encountered in distinguishing between different diseases having basically the same symptoms has been commented on by several authors [5,53,129]. Since isosclerone (**21**, Figure 16) and EPS are both produced by esca-associated fungi such as *Pa. chlamydospora*, *Pm. aleophilum* [54,55,58], *N. parvum* [74,119], and possibly other Botryosphaeriaceae species as well, it is necessary to clarify whether these toxins are related to the foliar symptoms of leaf stripe disease (young esca) or to BDA, if this disease is confirmed. If the symptoms cannot be distinguished, it would explain the confusion about how to diagnose these diseases if the diagnosis is based solely on the foliar symptoms. Although the cause of the chloro-necrotic foliar symptoms of esca still needs to be fully elucidated [5], it is possible to hypothesise that the foliar symptoms are the result of the synergistic action of the toxic metabolites produced by these pathogens, all of which colonise grapevine.

While isosclerone is produced by both the pathogenic botryosphaeriaceous fungi and the fungi causing esca, the melleins and their derivatives appear to be produced only by the botryosphaeriaceous fungi [125,127,130]. Tyrosol (**29**, Figure 16) is another toxic metabolite produced by plants and fungi, including *B. obtusa* [123,125,131].

The fact that *N. parvum* produce isosclerone and exopolysaccharides is very interesting not only because these compounds are also produced by *Pa. chlamydospora* but because they are also involved in causing foliar symptoms of young esca and grapevine leaf-stripe disease [58,103]. Although the exact cause of these chloro-necrotic foliar symptoms is still unclear [5], they could be the result, wholly or in part, of the synergistic action of the toxic metabolites produced by species of Botryosphaeriaceae and *Pa. chlamydospora* [132].

### 4.3. Toxins Produced by *Diplodia Seriata*

**Figure 17:**
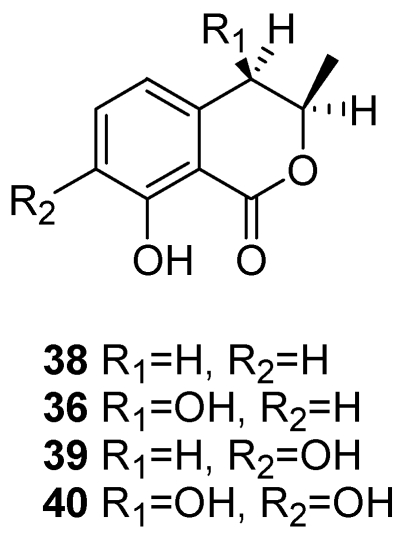
Structures of mellein, of (3*R*,4*R*)-(-)-4-hydroxymellein, of (3*R*)-7-hydroxymellein and of (3*R*, 4*R*)-*cis*-4,7-dihydroxymellein (**38, 36, 39, 40**), produced by *Botryosphaeria obtusa*.

*Diplodia seriata* (teleomorph: *Botryosphaeria obtusa*) is frequently associated with wood cankers of grapevine, sometimes named black dead arm or BDA [112,133], although this disease was initially reported by Lehoczky [134] as being caused not by *D. seriata* but by *D. mutila*. *D. seriata* also causes black rot of apple fruit, and produces several phenolic dihydroisocoumarins, such as mellein, *cis*-(3*R*,4*R*)-4-hydroxymellin and 5-hydroxymellein [125,128]. However, Djoukeng *et al*. [130] identified four phytotoxic compounds from a culture filtrate of *D. seriata* strain F-99-2, isolated from the vine cv. Cabernet Sauvignon. From an organic extract of the culture filtrates they isolated three known melleins, which on the basis of their spectroscopic data were identified as mellein, (3*R*,4*R*)-4-hydroxymellein, (3*R*)-7-hydroxymellein and (3*R*,4*R*)-4,7-dihydroxymellein (**38, 36 and 39**, Figure 17). Using the same technique they further isolated a unknown mellein, characterised as (3*R*,4*R*)-4,7-dihydroxymellein (**40**, Figure 17). A bioassay of vine cv. Gamay leaves found that (3*R*,4*R*)-4,7-dihydroxy-mellein was the most active metabolite, causing full leaf necrosis with a minimum inhibitory concentration (MIC) of 2 μg mL^−1^, although compounds **36, 38, and 39** (Figure 17) had a similar degree of toxicity with a MIC of 3 μg mL^−1^[130]. These authors using a HPLC DAD-MS analysis found that mellein (**38**, Figure 17) also occurred in infected vine wood inoculated with *D. seriata*. In that case, mellein would be a good diagnostic marker for *D. seriata* in diseased vines, and could be used to differentiate between esca and BDA at an early stage, since the melleins and their derivatives are not produced by esca-associated pathogens such as *Pa. chlamydospora*, *Pm. aleophilum* or *F. mediterranea* [130]. Mellein and its derivatives are also produced by many other non-botryosphaeriaceous fungi, including the genera *Aspergillus*, *Cercospora*, *Cryptosporiopsis*, *Hypoxylon*, *Phoma*, *Pezicula*, *Plectophomella*, *Septoria* and *Xylaria*, and they have phytotoxic, zootoxic and moderate antifungal activities [124,127].

As regards other toxins of *D. seriata*, it has been reported that tyrosol and *p*-hydroxybenzaldehyde were also produced by this pathogen, but only from an isolate obtained from apple [125,128]. Recently tyrosol has further been detected from *N. parvum* [119], and it cannot be excluded that some day *D. seriata* isolates will also be found to produce this toxin. 

## 5. Grouping in Different Chemical Families of Phytoxins Produced by Different Fungi

Frequently fungi produced toxins belonging to different classes of natural compounds and some of them are also produced by other fungi. However, a single toxin could be synthesized by one species and it would represents a taxonomical mark. A breakdown of the fungi and the toxins they produce is as follows:

*Pm. aleophilum* produces naphthalenone pentaketides and polyphenols; *Pa. chlamydospora:* naphthalenone pentaketides, polyphenols and anthraquinones; *E. lata:* acetylenic polyphenols, heterocyclic analogues and dihydro-γ-pyrones; and the Botryosphaeria species: naphthalenone pentaketides, melleins and polyphenols.

In these groupings the naphthalenone pentaketides and polyphenols are common to *Pm. aleophilum*, *Pa. chlamydospora* and Botryosphaeria species. Toxins produced (among others) by only one fungus, and which may serve to detect that fungus, are: the anthraquinones, found only in *Pa. chlamydospora*, the acetylenic phenols and dihydro-γ-pyrones, both specific to *E. lata*, and the melleins exclusive to the Botryosphaeriaceous species.

## 6. Conclusions

Of the several diseases affecting grapevine, trunk diseases caused by fungi are important since they cause devastating epidemics and also considerable annual yield losses. Studies on toxins produced by grapevine trunk pathogens have become more frequent in the last two decades, and one of the findings has been that most of these pathogens produce secondary metabolites that are toxic to plants. 

These phytotoxic substances have been at least in part chemically characterised, but their mode of action remains mostly to be elucidated. They have not yet been made the subject of particular study, except for eutypine, eutypinol, eulatinol and other metabolites produced by *E. lata* [32,33,34,35,38]. The toxicity of *E. lata* is probably due to a number of metabolites that are structurally related, each having a different level of toxicity and different molecular targets within the vine cell [32]. Of the toxins produced by the other trunk pathogens we know little that is significant (something is known only about the action of the naphthalenones, whose toxicity is thought to be linked to their oxidant properties). 

All the bioactive substances produced by *Pa. chlamydospora*, *Pm. aleophilum*, and the botryosphaeriaceous species were toxic to vines and other plants in toxicity tests on the leaves, calli or protoplasts of the host plants; when tested on the leaves of healthy plants at least some of these substances reproduced to a certain extent the original symptoms of the disease they caused [24,33,55,56,58,60,74,78]. These findings, though significant, are not generally thought to be sufficient to prove that these substances have a role in the diseases caused by the fungal pathogens; for now, such a conclusion would be mere speculation. 

Some phytotoxic metabolites, such as the EPSs, the naphtalenone pentaketides and the polyphenols are common to more than one fungus, whereas other metabolites (the anthraquinones, the acetylenic polyphenols and dihydro-γ-pyrones and the mulleins) are specific to an individual fungus. Since the main pathogens examined in this review produce both localised symptoms in the wood and also in some cases symptoms on the leaves, it can be hypothesised that the metabolites first act at the place in the wood where they are produced, causing darkening of the tissues, necrosis of the parenchyma cells surrounding the vessels, exudation of dark gum into the vessels, and occlusion of the xylem vessels, and that subsequently some of these metabolites also act at a distance from the place of production, since they accumulate in the leaves.

As regards the leaves, it has been mentioned that in vines affected with grapevine leaf stripe (one of the vascular syndromes in the esca complex), significant physiological changes take place in both the chlorotic portion and the surrounding green portion of the leaves with tiger-stripes, and even in the (still) asymptomatic leaves of those vine arms that also bear tiger-striped leaves. These physiological changes mainly impair photosynthesis, so that it seems reasonable to assume that one or more of the metabolites, including the EPSs, act directly or indirectly on the plant metabolism and on the functionality of the chloroplasts. As for the mode of action of EPSs, it has been hypothesised that their main effect is to plug the xylem vessels, but, since the EPSs also accumulate in the leaf mesophyll, a direct effect of these macromolecules on the chloroplasts cannot be excluded, either by the EPSs in their original form, or after they have been partially degraded, a process which reduces their molecular weight but increases their mobility.

As stated above, the chlorosis and necrosis of esca-affected leaves can also be interpreted as a premature senescence resulting, at least in part, from the peroxidation of the membrane lipids [56], as shown by the increase in anthocyanin levels caused by the toxic polypeptides produced by *Pa. chlamydospora* [78]. An increase in the anthocyanin levels of vine leaves is also a first stage in the formation of tiger stripes, and it is clear that the fully formed leaf stripe symptoms resemble senescent leaves about to fall. 

Toxin levels and the rate of increase of these levels in the leaves may also explain some other characteristics of grapevine trunk diseases, particularly why leaf symptoms vary over time and over space. It is well known that vines affected with esca do not all begin to show foliar symptoms at the same time in a growing season. Foliar symptoms generally appear in June-July, but they can also appear in May, or in August-September, and in some infected vines they do not appear at all, or not on all leaves, for one or even more growing seasons. With these infected but temporarily asymptomatic vines it is not possible to predict in what growing season any of these vines, or any portion of their vine crown, will again begin to show leaf symptoms. *E. lata* dieback and Botryosphaeriaceae species share some of the same peculiarities. While all biological phenomena are naturally subjected to variation, it seems that with these diseases, at least some of the reactions recorded were due to the type and the particular mixture of the toxins that accumulated in the leaves, and this in turn was partly the result of the physiological status of the vine and the environmental conditions.

As regards the basidiomycetes, common agents of wood rot in grapevine (*F. mediterranea* in Europe and the Mediterranean area; other basidiomycetes in other vine growing areas), the main virulence factors of these fungi consist in their degrading enzymes. However, recent studies have shown that these fungi also produce toxic metabolites *in vitro.* The role of these toxins *in planta* during symptom development remains to be elucidated [80].

In conclusion, some of the studies discussed in this review suggest that toxic metabolites have a notable role in causing the symptoms of grapevine trunk diseases. However, the exact mode of operation of these toxins, and the way in which they contribute, individually or jointly, to the expression of symptoms, is still not well understood. Further research is needed to elucidate the interaction between the phytotoxins and other abiotic factors which are involved in disease development, such as rainfall and the temperature regime [44,135].
